# Organ procurement organizations Internet enrollment for organ donation: Abandoning informed consent

**DOI:** 10.1186/1472-6939-7-14

**Published:** 2006-12-22

**Authors:** Sandra Woien, Mohamed Y Rady, Joseph L Verheijde, Joan McGregor

**Affiliations:** 1 From the Bioethics, Policy, and Law Program, School of Philosophy and Life Sciences, Arizona State University, Tempe, Arizona, USA; 2 Department of Critical Care Medicine, Mayo Clinic Hospital, Phoenix, Arizona, USA; 3 Physical Medicine and Rehabilitation, Mayo Clinic Hospital, Phoenix, Arizona, USA

## Abstract

**Background:**

Requirements for organ donation after cardiac or imminent death have been introduced to address the transplantable organs shortage in the United States. Organ procurement organizations (OPOs) increasingly use the Internet for organ donation consent.

**Methods:**

An analysis of OPO Web sites available to the public for enrollment and consent for organ donation. The Web sites and consent forms were examined for the minimal information recommended by the United States Department of Health and Human Services for informed consent. Content scores were calculated as percentages of data elements in four information categories: donor knowledge, donor consent reinforcement, donation promotion, and informed consent.

**Results:**

There were 60 Web sites for organ donation enrollment serving the 52 states. The median percent (10 percentile-90 percentile) content scores of the Web sites for donor knowledge, donor consent reinforcement, and donation promotion were 33% (20–47), 79% (57–86), and 75% (50–100), respectively. The informed consent score was 0% (0–33). The content scores for donor knowledge and informed consent were significantly lower than donor consent reinforcement and donation promotion for all Web sites (*P *< .05). The content scores for the four categories were similar among the 11 regions of the United Network for Organ Sharing.

**Conclusion:**

The Web sites and consent forms for public enrollment in organ donation do not fulfill the necessary requirements for informed consent. The Web sites predominantly provide positive reinforcement and promotional information rather than the transparent disclosure of organ donation process. Independent regulatory oversight is essential to ensure that Internet enrollment for organ donation complies with legal and ethical standards for informed consent.

## Background

Recent advances in transplantation have expanded the criteria of age and end organ diseases for organ recipients thus exponentially increasing the waiting list for new organs[1,2]. The expanded pool of recipients has increased the demand for the donation and use of deceased organs [3]. The Health Resources and Services Administration (HRSA) of the United States Department of Health and Human Services (DHHS) has introduced the Organ Donation Breakthrough Collaborative to address the evolving crisis of transplantable organs shortage [4]. The charge of the Breakthrough Collaborative is to rapidly enable organ procurement organizations (OPOs) to increase deceased organ donation and utilization rates in the community. Fifty eight OPOs are designated by the United States Centers for Medicare and Medicaid Services (CMS) to provide donor services within defined geographic areas for the 52 states (including the territories of Guam and Puerto Rico). Each OPO operates within a designated donation service area (DSA) and acts as a conduit between organ donor hospitals and transplant centers within a part of a state, a whole state, or multiple states [5,6]. OPOs are also responsible for approaching potential donors and families to discuss the option of deceased organ donation and for coordinating the recovery, preservation, and transportation of organs donated for transplantation. OPOs have used the Internet as an effective and efficient portal into the community to encourage registration for deceased organ donation and to accomplish the goals of the Breakthrough Collaborative [7].

CMS, federal agencies and Joint Commission on Accreditation of Healthcare Organizations introduced regulations for participation of all hospitals across the United States in non-heart beating (also referred to as cardiac or imminent death) organ donation [8-11]. With mandatory participation, it is predicted that organs after cardiac or imminent death rather than brain-death will be the main source of procurable organs for transplantation. Organ procurement from cardiac or imminent death donors, however, deviates from the practice adopted for brain-death donors. Cardiac and imminent death donation protocols require interventions to be initiated before the donor has been declared dead. In response to the sharp transition toward cardiac or imminent death organ donation, this study was designed to examine the Web sites and consent forms of OPOs that are available to persons contemplating organ donation after death. We examined whether the public has been provided with sufficient knowledge about the organ donation process and the differences between the procedures for brain-death, cardiac, and imminent death donation to enable them to make an informed decision for donation consent.

## Methods

The study was approved by the institutional review boards of the Mayo Foundation and Arizona State University.

### Selection of Web sites

Ninety one OPO Web sites links were obtained from the alphabetical listing of the 52 states [12]. Seventeen Web sites were shared by more than one state. The eleven regions of United Network for Organ Sharing (UNOS) were covered by 59 Web sites [6]. In addition, the Web site of Donate Life America available for all states was also included in the study [see additional file 1].

### Data elements and extraction

**Figure 1:**
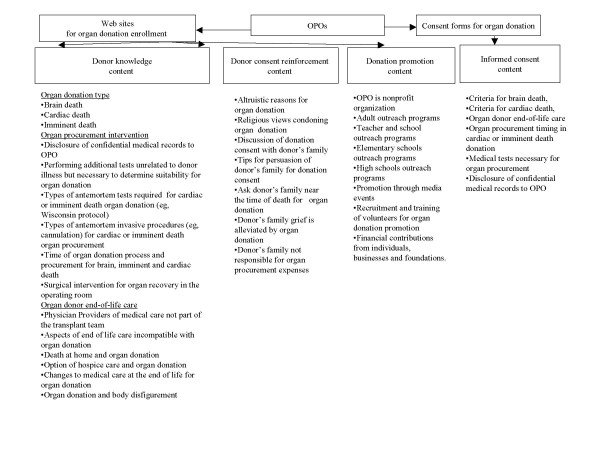
The study design of informational content and consent forms on the sixty identified Web sites.

The information content of the Web sites and consent forms were examined using the data elements listed in Figure 1. Analysis of the content of these web sites classified the information into four categories: 1) donor knowledge content, 2) donor consent reinforcement content, 3) donation promotion content, and 4) informed consent content. The data elements were developed from the recommendations published by the United States DHHS Advisory Committee on Organ Transplantations (ACOT) and the Office of the Assistant Secretary for Planning and Evaluation as minimal requirements for informed consent for organ donation [8,9,13]. The Web sites were accessed between 2 May, 2006 to 1 June, 2006 for data collection. The consent forms were accessed between 12 June, 2006 to 15 June, 2006. All the consent forms were printed out from the Web sites. The Web sites and consent forms were analyzed for the presence of the minimal information required for informed consent for organ donation (Figure 1). Each individual Web site including its links and affiliated organ donation registry was searched using Microsoft Internet Explorer Version 6.0 (Microsoft Corp., Redmond, WA). A data element was considered present if the data element was mentioned or referred to on the Web site or its links to other Web pages.

### Data entry and analysis

Each data element was recorded into an Excel spreadsheet with binary numeric input (present = 1, absent = 0). The content scores were calculated as the sum of data elements present and expressed as a percentage of the total in each category. The category for the donor knowledge score (15 data elements) included description of organ donation types, organ procurement medical interventions, and organ donor end-of-life care. The category for the donor consent reinforcement score included 7 data elements. The category for the donation promotion score included 8 data elements. Finally, the category for the informed consent score for the printed consent forms included 6 data elements. Interobserver agreement for data abstraction was good (Kappa statistics 0.8, P < 0.01). Statistical analysis was performed using JMP Statistical software (version 5.1; SAS Institute Inc., Cary, NC, USA).

## Results

Sixty Web sites for organ donations were identified and evaluated [see additional file 1]. Twenty four states (46%) had two or more OPO Web sites. Thirty six Web sites (60%) were linked to state registries for consent to organ donation; the other 24 Web sites directed site visitors to fill out state donor cards or include signed consent on the driver licenses' issued by the Department of Motor Vehicle (DMV). Table 1 describes the information content of the Web sites and consent forms. The median percent (10 percentile-90 percentile) content scores for donor knowledge 33% (20–47) and informed consent 0% (0–33) scores were significantly (*P *< .05) lower than donor consent reinforcement 79% (57–86) and donation promotion 75% (50–100) scores.

**Table 1:** Information content of organ procurement organizations Web sites encouraging organ donation enrollment.

Data element	N = 60(%)*	Confidence interval ^+^
**Organ donation type**		
Brain death	54(90)	82–95
Cardiac death	30(50)	41–62
Imminent death	6(10)	5–18
**Organ procurement medical intervention**		
Disclosure of confidential medical records to OPO	15(25)	18–37
Performing additional tests unrelated to donor illness but necessary to determine suitability for organ donation	27(45)	35–56
Types of antemortem tests required for cardiac or imminent death organ donation (eg, Wisconsin protocol)	1(2)	0–7
Types of antemortem invasive procedures (eg, cannulation) required for cardiac or imminent death organ procurement	1(2)	0–7
Time of organ donation process and procurement for brain, imminent, and cardiac death	1(2)	0–7
Surgical intervention for organ recovery in the operating room	52(87)	78–92
**Organ donor end-of-life care**		
Physicians Providers of medical care not part of the transplant team	60(100)	95–100
Aspects of end-of-life care incompatible with organ donation	0	0
Death at home and organ donation	5(8)	4–16
Option of hospice care and organ donation	0	0-0
Changes to medical care at the end of life for organ donation	0	0-0
Organ donation and body disfigurement	59(98)	93–100
**Donor knowledge score, %**	33	20–47
**Donor consent reinforcement**		
Altruistic reasons for organ donation	59(98)	93–100
Religious views condoning organ donation	58(97)	90–99
Discussion of donation consent with donor's family	60(100)	96–100
Tips for persuasion of donor's family for donation consent	9(15)	9–24
Ask donor's family near the time of death for organ donation	55(92)	84–95
Donor's family grief is alleviated by organ donation	26(43)	33–53
Donor's family not responsible for organ procurement expenses	60(100)	96–100
**Donor consent reinforcement score, %**	79	57–86
**Donation promotion**		
OPO is nonprofit organization	52(87)	78–92
Adult outreach programs	59(98)	93–100
Teacher and school outreach programs	52(87)	78–92
Elementary schools outreach programs	17(28)	20–39
High schools outreach programs	36(60)	49–70
Promotion through media events	44(73)	63–81
Recruitment and training of volunteers for organ donation promotion	56(93)	86–97
Financial contributions from individuals, businesses, and foundations	33(55)	44–65
**Donation promotion score, %**	75	50–100
**Consent forms**		
Criteria of brain death	0	0
Criteria of cardiac death	0	0
Organ donor end-of-life care	0	0
Organ procurement timing in cardiac or imminent death donation	0	0
Medical tests necessary for organ procurement	13(22)	14–32
Disclosure of confidential medical records to OPO	9(15)	9–24
**Informed consent score, %**	0	0–33

* Values are numbers or median (percent)

^+ ^Range (10% percentile to 90% percentile).

OPO = Organ Procurement Organization.

Of the 52 states, 26 (50%) had a donor registry and the rest had donor cards or DMV-based consent for organ donation. No consent form in any state disclosed brain and cardiac death criteria for donation, organ donor end-of-life care, or organ procurement timing after cardiac death. The consent forms disclosed medical tests necessary for organ procurement in 9 states (17%) and informed potential donors that their confidential medical records would be given to OPOs in 8 states (15%).

**Figure 2:**
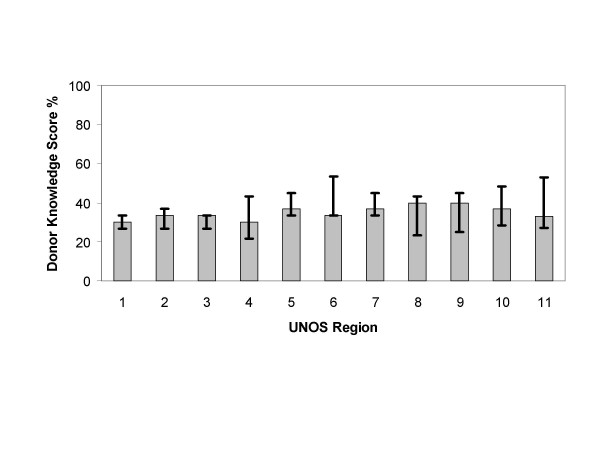
Median donor knowledge scores of Web sites established by organ procurement organizations within the 11 United Network for Organ Sharing (UNOS) regions in the United States. The error bars show interquartile ranges.

**Figure 3:**
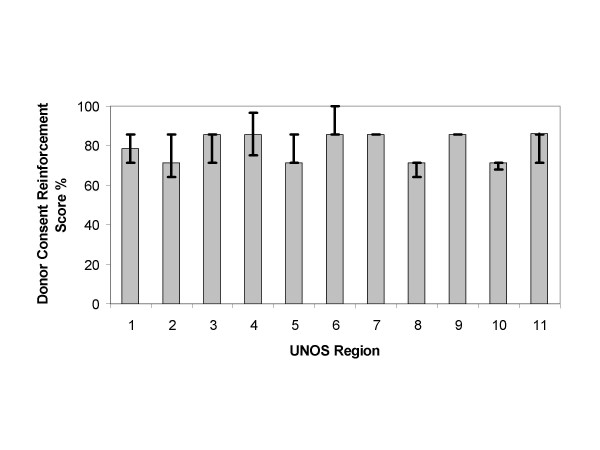
Median donor consent reinforcement scores of Web sites established by organ procurement organizations within the 11 United Network for Organ Sharing (UNOS) regions in the United States. The error bars show interquartile ranges.

**Figure 4:**
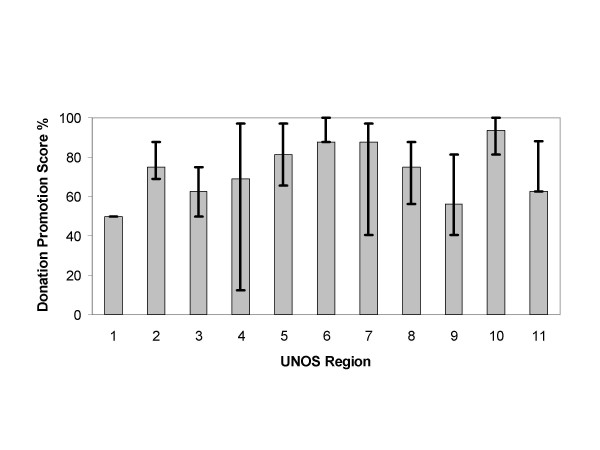
Median donation promotion scores of Web sites established by organ procurement organizations within the 11 United Network for Organ Sharing (UNOS) regions in the United States. The error bars show interquartile ranges.

**Figure 5:**
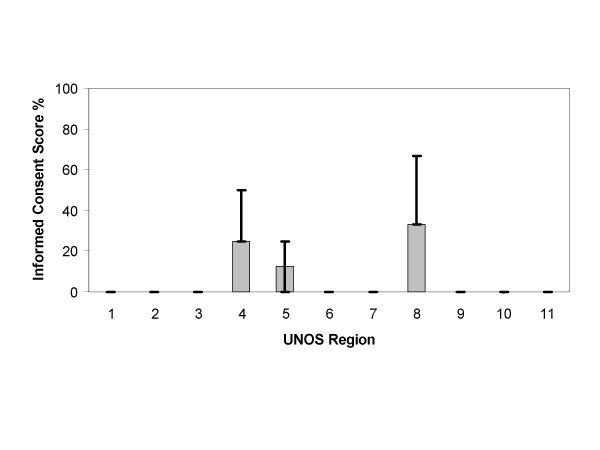
Median informed consent scores of Web sites established by organ procurement organizations within the 11 United Network for Organ Sharing (UNOS) regions in the United States. The error bars show interquartile ranges.

Analysis of the content scores of the Web sites and consent forms by UNOS region are depicted in Figures 2, 3, 4, 5. The content scores for donor knowledge (Fig. 2) and informed consent (Fig. 5) were equally low for all UNOS regions. The content scores for donor consent reinforcement (Fig. 3) and donation promotion (Fig. 4) were equally high for all UNOS regions.

## Discussion

Our findings showed that the disclosure on OPO Web sites and in online consent forms lacked pertinent information required for informed enrollment for deceased organ donation. In stead, the information content of these Web sites concentrated on providing positive reinforcement to consent and on promoting organ donation.

### The role of the Internet in organ donation

The Internet is a powerful and effective tool used by the transplantation community to increase public awareness of organ shortage and to appeal for organ donation. Most OPOs currently still focus on the face to face consent process, including experienced professionals or organ requesters leading the discussion for organ donation. The OPOs are increasingly referring to organ donation registries which are linked to DMV driver license or Internet registration to make the intent for donation legally binding [14,15]. In the 2006 report, the Committee on Increasing Rates of Organ Donation of the Institute of Medicine (IOM) has encouraged the universal adoption of organ donor registries across the United States to increase the rate of donation consent [13,16]. The organ donor registries are linked to OPOs Web sites to facilitate and expedite electronic organ donor registration.

Nowadays, the Internet is used to increase online consent for organ donor registry or cards across the United States [7]. The informational content of OPO Web sites and consent forms is quickly becoming an integral component in the individual decision to consider organ donation. Our findings show that the informational content available to the general public on these Web sites reinforces positive attitudes about organ donation and promotes donation-related activities. The Web sites and consent forms, however, lack basic factual knowledge for the potential donor on essential aspects of the organ donation process. The absence of such essential information raises serious doubts about whether potential donors are truly informed at the time of consent.

### Standards for disclosure on the Internet

Legal, ethical, and medical standards exist for informed consent in medical practice [8,9,17]. At a minimum, standards for informed consent require disclosure of all relevant information necessary for that person to make an informed decision based on personal values and preferences. Relevant information must encompass the nature of the procedure with its potential risks and benefits, any procedure-related protocols, alternative options, and related outcomes for the individual. The legal responsibility of the medical profession for informed consent is derived from the ethical standards for ensuring autonomous decision making. Full disclosure of material aspects of the procedure must also be presented in a manner that enables persons to understand the consequences of the decision they must make. The medical requirements for informed consent enforce the core bioethical principles of autonomy and beneficence.

Visitors to the OPO Web sites are invited to sign up as organ donors through state registries or donor cards in a process that will constitute a general consent for organ donation. The official stance of both DHHS and the IOM is that certain minimal requirements for disclosure should be met prior to organ donation consent [8,9,16]. Yet, the information content on the OPO Web sites and consent forms does not meet these requirements.

The salient differences between the process of organ donation after cardiac or imminent death and organ donation after brain death have not been emphasized in the public domain since its integration into transplantation practice [18]. We have expressed concern that consent forms for organ donation do not disclose or distinguish between brain and cardiac death criteria and processes. It can be argued that this information is improper to disclose at the time of registration for organ donation. The argument can be made that the disclosure of types of organ donation should be the domain of the health care professionals involved with the potential donor during the time leading to the declaration of death. However, the current practice and federal guidelines designate the OPOs and affiliates rather than the health care professionals to explain and obtain consent for different types of organ donation [8,9,15]. Therefore, the OPOs have the primary responsibility for the disclosure of information pertaining to the types of organ donation in order for the donors and families to make informed choices. The President's Council on Bioethics have expressed concerns similar to ours that certain issues pertinent to cardiac or imminent death organ donation have not been addressed explicitly by hospitals and OPOs in their donation consent process and protocols or by those bodies that have made recommendations for reforming or expanding deceased organ donation practice [17].

Our findings show that OPO Web sites do not delineate relevant and essential aspects of cardiac or imminent death organ donation. The process for organ donation starts *before *the declaration of death because death is *anticipated *soon after removal of life support in persons approaching cardiac or imminent death donation. In brain death, the organ donation process begins *after *the declaration of death. Clarification of the timing difference between the two possible alternatives of organ donation, and specifically the procurement time after a declaration of cardiac death, are not delineated on the OPO Web sites.

Certain non-beneficial antemortem testing [19] and procedures for organs preservation [11,20] that are performed on the donor before procurement are also not clearly stated on these Web sites. On the other hand, certain beneficial aspects of donor end-of-life care may be compromised[21]. Few Web sites have disclosed how out-of-hospital death e.g. at home or in a hospice setting may be influenced by cardiac or imminent death organ donation. For out-of-hospital cardiopulmonary arrest, preparation for organ donation has been added to standard resuscitation protocols with cooling to sustain organs viability during transportation to hospitals for surgical recovery of the desired organs[16].

None of the Web sites disclosed how the organs preservation procedures crucial for successful procurement can interfere with certain quality indicators for end-of-life care. Interestingly, The Robert Wood Johnson Foundation Critical Care End-of-Life Peer Workgroup has developed and recommended compliance with certain quality indicators to ensure that end-of-life care is *not *sacrificed for the purpose of organ donation [22]. The Critical Care Peer Workgroup of the Promoting Excellence in End-of-Life Care Project has reported wide variability and prevalence of deficiencies in end-of-life care across the United States [23]. The workgroup reported over 75% of the surveyed intensive care units did not monitor the quality of end-life-care. It is not surprising that the end-of-life care metrics have neither been measured nor reported in organ donors [21]. The President's Council on Bioethics has re-affirmed that there are obligations to disclose how the organ donor's end-of-life care will change as a result of the decision to donate and there is an ethical imperative to disclose the trade-off for a true informed consent [17].

### Presumed consent or mandated choice

The Uniform Anatomical Gift Act (UAGA) of 1968 (amendment in 1987 and revision in 2006) specified that the donor's authorization to donate as recorded on an organ donor card, on the individual's driver's license, or in a donor registry is as legally binding as an advance directive regarding end-of-life care [15]. The revised UAGA in 2006 has assigned explicit priority to the donor's intent so that the donor consent for organ donation becomes irrevocable and does not require consent or concurrence of any person after the donor's death[24]. In compliance with the UAGA legislation, the current OPO practice is to proceed with organ donation with a pre-signed organ donor card or registry without requiring family consent in nineteen jurisdictions within the United states [24,25]. The UAGA amendment has also enabled OPOs to procure organs even with family refusal to donation if the donor has documented their intent to donate [15]. However, the application of UAGA also demands that voluntary consent of the organ donor is a transparent process.

The Committee on Increasing Rates of Organ Donation of the IOM has debated consent options for deceased organ donation [16]. Presumed consent has been considered a favorable option for organ donation. Currently, state organ donation laws require individuals to decide on becoming organ donors, and the default option in the absence of express consent, is nondonation. Within the presumed consent model, the default option is replaced, in the absence of express rejection, with donation. Therefore, in the absence of an individual's express decision, the individual's consent rather than refusal for organ donation will be presumed. The IOM has supported the concept of presumed consent and proposes that future legislative enactment can increase organ donors pool[16]. Another consent option is the mandated choice model which requires each individual to choose whether or not to be an organ donor. The latter option will open public and societal access to information on the process of organ donation and also will demand understanding of the relayed information. States will have to enact legislation that requires individuals either to opt in or opt out of organ donation. The advantage of a mandated choice model over the presumed consent model is that the mandated choice model fortifies the moral requirement of true informed consent with regard to organ donation. However, the IOM has recommended against future legislative enactment of the mandated choice because it can potentially decrease the organ donors pool. The IOM prefers presumed consent to increase the rate of organ donation because that type of consent does not require the development of costly public education programs necessary for the implementation of a mandated choice[16].

### Study implications

The increased pressure for organ donation registration raises serious concerns whether the current information disseminated by the OPOs' Web sites satisfy the legal and ethical requirements of informed consent. The ACOT, OPOs and transplantation community organizations have long been the advocates of the critical aspect of consent for organ donation[6,9]. The inherent interest of the above entities to promote organ donation has created sufficient conflict to introduce self-serving bias in information disclosure to the public and organ donors. Therefore, we recommend that in order to maintain transparency and public trust, an independent entity with no potential for conflict of interest should take charge of the process of enrollment for organ donation. The independent entity can take charge of the public eduction in the community and determine the ethical and legal standards required for disclosure of information before registration of organ donation consent.

### Study limitations

The data collection on visited Web sites and consent forms were time sensitive. The possibility that certain data elements might have been changed since the survey completion could influence the reported content. Certain data elements were embedded within the Web sites and therefore, could be easily missed or less readily available to the public visiting these sites. For instance, several Web sites included critical information on brain death, cardiac and imminent death in the health professionals section rather than under the heading of donors' information. While this information was still counted as disclosed in this study, there was a possibility that visitors to the Web sites would miss the information because of poor accessibility. The study did not address the differential accessibility of the information categories (i.e. donor knowledge, consent reinforcement, promotion) on the Web sites to visitors. The accessibility to information within the Web sites could influence the organ enrollment process.

## Conclusion

The Web sites and consent forms for public enrollment in organ donation do not fulfill the necessary requirements for informed consent. The Web sites predominantly provide positive reinforcement and promotional information rather than the transparent disclosure of organ donation process. Independent regulatory oversight is essential to ensure that Internet enrollment for organ donation complies with legal and ethical standards for informed consent.

## List of abbreviations

ACOT = Advisory Committee on Organ Transplantations

CMS = Centers for Medicare and Medicaid Services

DHHS = Department of Health and Human Services

DMV = Department of Motor Vehicle

DSA = Donation Service Area

HRSA = Health Resources and Services Administration

OPO(s) = Organ Procurement Organization(s)

UAGA = Uniform Anatomical Gift Act

UNOS = United Network for Organ Sharing

## Competing interests

There are no affiliations or financial involvement with any organization or entity with a direct financial interest in the subject matter or materials discussed in the manuscript. Authors have no financial or non-financial competing interests to disclose.

## Authors' contributions

The authors (SW, MYR, JLV and JM) attest they have made substantial contributions to conception and design, or acquisition of data, or analysis and interpretation of data; that they have been involved in drafting the manuscript or revising it critically for important intellectual content; that they have given final approval of the version to be published; and that they have participated sufficiently in the work to take public responsibility for appropriate portions of the content. SW, MYR, JLV and JM had full access to all of the data in the study and take responsibility for the integrity of the data and the accuracy of the data analysis. SW, MYR, JLV and JM read and approved the final manuscript.

## Pre-publication history

The pre-publication history for this paper can be accessed here:



## Supplementary Material

Additional File 1 Web sites encouraging organ donation enrollment. The Web sites encouraging organ donation enrollment were accessed between 2 May, 2006 to 1 June, 2006 and 12 June, 2006 to 15 June, 2006. Click here for file

## References

[B1] Futagawa Y, Terasaki PI, Waki K, Cai J, Gjertson DW (2006). No Improvement in Long-Term Liver Transplant Graft Survival in the Last Decade: An Analysis of the UNOS Data. American Journal of Transplantation.

[B2] Shiffman ML, Saab S, Feng S, Abecassis MI, Tzakis AG, Goodrich NP, Schaubel DE (2006). Liver and Intestine Transplantation in the United States, 1995-2004. American Journal of Transplantation.

[B3] Delmonico FL, Sheehy E, Marks WH, Baliga P, McGowan JJ, Magee JC (2005). Organ donation and utilization in the United States, 2004. American Journal of Transplantation.

[B4] Marks WH, Wagner D, Pearson TC, Orlowski JP, Nelson PW, McGowan JJ, Guidinger MK, Burdick J (2006). Organ Donation and Utilization, 1995-2004: Entering the Collaborative Era. American Journal of Transplantation.

[B5] United Network for Organ Sharing United Network for Organ Sharing.. http://www.unos.org/.

[B6] Organ Procurement and Transplantation Network Organ Procurement and Transplantation Network. http://www.optn.org.

[B7] Merion RM, Vinokur AD, Couper MP, Jones EG, Dong Y, Wimsatt M, Warren J, Katz S, Leichtman AB, Beyersdorf T (2003). Internet-based intervention to promote organ donor registry participation and family notification. Transplantation.

[B8] Centers for Medicare & Medicaid Services- Department of Health and Human Services (2006). Medicare and Medicaid Programs; Conditions for Coverage for Organ Procurement Organizations (OPOs); Final Rule. 42 CFR Parts 413, 441, 486 and 498.. Federal Register.

[B9] U.S. Department of Health and Human Services Advisory Committee on Organ Transplantation Consensus Recommendations to the HHS Secretary.. http://www.organdonor.gov/acot.html.

[B10] Joint Commission on Accreditation of Healthcare Organizations(JCAHO) (2006). Revisions to Standard LD.3.110.. Jt Comm Perspect.

[B11] Bernat JL, D'Alessandro AM, Port FK, Bleck TP, Heard SO, Medina J, Rosenbaum SH, Devita MA, Gaston RS, Merion RM, Barr ML, Marks WH, Nathan H, O'Connor K, Rudow DL, Leichtman AB, Schwab P, Ascher NL, Metzger RA, Mc Bride V, Graham W, Wagner D, Warren J, Delmonico FL (2006). Report of a National Conference on Donation after cardiac death. American Journal of Transplantation.

[B12] OPOs in the United States OPOs in the United States.. http://www.transweb.org/reference/maps/opo_image_map/alphalist.htm.

[B13] U.S. Department of Health and Human Services (DHHS) Office of the Assistant Secretary for Planning and Evaluation Analysis of State Actions Regarding Donor Registries.. http://www.organdonor.gov/aspehealth.html.

[B14] Association of Organ Procurement Organizations AOPO Position Statement on A National Registry Network.. http://www.aopo.org/aopo/index.asp.

[B15] National Conference of Commissioners on Uniform State Laws Revised Uniform Anatomical Gift Act (2006).. http://www.law.upenn.edu/bll/ulc/uaga/2006final.htm.

[B16] Childress JF, Liverman CT, Committee on Increasing Rates of Organ Donation-Board on Health Sciences Policy-Institute of Medicine (2006). Organ Donation: Opportunities for Action..

[B17] Rubenstein A, Cohen E, Jackson E The Definition of Death and the Ethics of Organ Procurement from the Deceased. The President's Council on Bioethics.. http://www.bioethics.gov/background/rubenstein.html.

[B18] DeVita MA, Snyder JV (1993). Development of the University of Pittsburgh Medical Center policy for the care of terminally ill patients who may become organ donors after death following the removal of life support. Kennedy Institute of Ethics Journal.

[B19] Lewis J, Peltier J, Nelson H, Snyder W, Schneider K, Steinberger D, Anderson M, Krichevsky A, Anderson J, Ellefson J, D'Alessandro A (2003). Development of the University of Wisconsin donation after cardiac death evaluation tool. Progress in Transplantation.

[B20] United Network for Organ Sharing United Network for Organ Sharing-Donor Management Critical Pathway.. http://www.unos.org/resources/donorManagement.asp?index=2.

[B21] Rady MY, Verheijde JL, McGregor J (2006). Organ donation after circulatory death: the forgotten donor. Critical Care.

[B22] Clarke EB, Curtis JR, Luce JM, Levy M, Danis M, Nelson J, Solomon MZ, Robert Wood Johnson Foundation Critical Care End-Of-Life Peer Workgroup Members (2003). Quality indicators for end-of-life care in the intensive care unit.. Crit Care Med.

[B23] Nelson JE, Angus DC, Weissfeld LA, Puntillo KA, Danis M, Deal D, Levy MM, Cook DJ, for the Critical Care Peer Workgroup of the Promoting Excellence in End-of-Life Care Project (2006). End-of-life care for the critically ill: A national intensive care unit survey. Crit Care Med.

[B24] The HHS Secretary's Advisory Committee on Organ Transplantation State Law and OPO Practice. State Laws on Adoption of the 1987 Amendments for Anatomical Gifting.. http://www.organdonor.gov/acotapp6.html.

[B25] Wendler D, Dickert N (2001). The consent process for cadaveric organ procurement: how does it work? How can it be improved?. JAMA.

