# Supplementary material for: Organ procurement organizations Internet enrollment for organ donation: Abandoning informed consent
Source: BMC Med Ethics. 2006 Dec 22;7:14. doi: 10.1186/1472-6939-7-14 (PMC1764895; doi:10.1186/1472-6939-7-14)
Supplement: Additional File 1 — Web sites encouraging organ donation enrollment. The Web sites encouraging organ donation enrollment were accessed between 2 May, 2006 to 1 June, 2006 and 12 June, 2006 to 15 June, 2006. [file 1472-6939-7-14-S1.doc]

**Web sites encouraging organ donation enrollment.**

| **Alphabetical list of OPO regions by states** | **Web Site URL** |
| --- | --- |
| Alabama Organ Center | http://www.uab.edu/aoc |
| Arkansas Regional Organ Recovery Agency | http://www.arora.org/ |
| California Transplant Donor Network | http://www.ctdn.org/ |
| California Transplant Donor Network  (Reno, Nevada) | http://www.ctdn.org/ |
| Carolina Donor Services | http://www.carolinadonorservices.org/ |
| Carolina Organ Procurement Agency  (Pittsylvania County, Virginia) | http://www.lifepoint-sc.org/ |
| Center for Donation and Transplant | http://www.timesunion.com/communities/cdt/ |
| Center for Organ Recovery & Education | http://www.core.org/ |
| Center for Organ Recovery & Education, CORE  (Southern New York) | http://www.core.org/ |
| Donor Alliance, Inc. | http://www.donoralliance.org/ |
| Donor Network of Arizona | http://www.dnaz.org/ |
| Finger Lakes Donor Recovery Network | http://www.donorrecovery.org/ |
| Gift of Life Donor Program  (formerly Delaware Valley Transplant Program) | http://www.donors1.org/ |
| Gift of Life Donor Program  (formerly Delaware Valley Transplant Program) (Central and Eastern Pennsylvania) | http://www.donors1.org/ |
| Gift of Life Donor Program  (formerly Delaware Valley Transplant Program) (Southern New Jersey) | http://www.donors1.org/ |
| Golden State Donor Services | http://www.gsds.org/ |
| Indiana Organ Procurement Organization, Inc. | http://www.iopo.org/ |
| Intermountain Donor Services | http://www.idslife.org/ |
| Intermountain Organ Recovery Systems  (Elko, Nevada) | http://www.idslife.org/ |
| Intermountain Organ Recovery Systems  (Southeastern Idaho) | http://www.idslife.org/ |
| Intermountain Organ Recovery Systems  (Western Wyoming) | http://www.idslife.org/ |
| Iowa Donor Network | http://www.iadn.org/ |
| Kentucky Organ Donor Affiliates | http://www.kyorgandonor.org/ |
| Life Connection of Ohio | http://www.lifeconnectionofohio.org/ |
| LifeBanc | http://www.lifebanc.org/ |
| LifeCenter Northwest | http://www.lcnw.org/ |
| LifeCenter Northwest (Northern Idaho) | http://www.lcnw.org/ |
| LifeGift Organ Donation Center | http://www.lifegift.org/ |
| LifeLink of Florida | http://www.lifelinkfound.org/Florida/fl.html |
| LifeLink of Georgia | http://www.lifelinkfound.org/georgia/ga.html |
| LifeLink of Georgia  (Aiken and Edgefield Counties in South Carolina) | http://www.lifelinkfound.org/georgia/ga.html |
| LifeLink of Puerto Rico | http://www.lifelinkfound.org/ |
| LifeLink of Southwest Florida | http://www.lifelinkfound.org/sw/sw.html |
| LifeNet | http://www.lifenet.org/ |
| LifePoint | http://www.lifepoint-sc.org/ |
| LifeShare of the Carolinas | http://www.lifesharecarolinas.org/ |
| LifeSource, Upper Midwest Organ Procurement  Organization, Inc. | http://www.life-source.org |
| Lifeline of Ohio Organ Procurement Agency, Inc. | http://www.lifelineofohio.org/ |
| Lifesharing Community Organ and Tissue  Donation | http://www.lifesharing.org/ |
| Louisiana Organ Procurement Agency | http://www.lopa.org/ |
| Mid-America Transplant Services | http://www.mts-stl.org/ |
| Mid-South Transplant Foundation, Inc. | http://www.midsouthtransplant.org/ |
| Mid-South Transplant Foundation, Inc.  (Eastern Arkansas) | http://www.midsouthtransplant.org/ |
| Mid-South Transplant Foundation, Inc.  (Northern Mississippi) | http://www.midsouthtransplant.org/ |
| Midwest Organ Bank, Inc. | http://www.mwob.org/ |
| Midwest Transplant Network | http://www.mwob.org/ |
| Mississippi Organ Recovery | http://www.msora.org/ |
| Nebraska Organ Retrieval System, Inc. | http://www.nedonation.org/ |
| Nebraska Organ Retrieval System, Inc.  (Western Iowa) | http://www.nedonation.org/ |
| Nevada Donor Network, Inc. | http://www.nvdonor.org/ |
| New England Organ Bank | http://www.neob.org/ |
| New Mexico Donor Services | http://www.donatelifenm.org/ |
| New York Organ Donor Network, Inc. | http://www.nyodn.org/ |
| NorthEast Organ Procurement Organization  (Western Massachusetts) | http://www.lifechoiceopo.org/ |
| NorthEast Organ Procurement Organization and  Tissue Bank | http://www.lifechoiceopo.org/ |
| Ohio Valley LifeCenter | http://www.lifecnt.org/ |
| Ohio Valley LifeCenter (Northern Kentucky) | http://www.lifecnt.org/ |
| Ohio Valley LifeCenter (Southeastern Indiana) | http://www.lifecnt.org/ |
| Oklahoma Organ Sharing Network | http://www.lifeshareoklahoma.org/ |
| OneLegacy Transplant Donor Network | http://www.onelegacy.org/ |
| Organ Donor Center of Hawaii | http://www.organdonorhawaii.com/ |
| Organ Procurement Organization at the University  of Florida | http://www.lifequestfla.org |
| Pacific NW Transplant Bank | http://www.pntb.org/ |
| Regional Organ Bank of Illinois, Inc. | http://www.giftofhope.org/ |
| Regional Organ Bank of Illinois, Inc.  (Northwest Indiana) | http://www.giftofhope.org/ |
| Southwest Transplant Alliance | http://www.organ.org/ |
| Tennessee Donor Services | http://www.dcids.org/ |
| Texas Organ Sharing Alliance | http://txorgansharing.org/ |
| The Sharing Network Organ Tissue Donation  Services | http://www.sharenj.org/ |
| TransLife/Florida Hospital | http://www.translife.org/ |
| Transplant Resource Center of Maryland | http://www.mdtransplant.org/ |
| Transplantation Society of Michigan | http://www.giftoflifemichigan.org/ |
| University of Miami OPO | http://surgery.med.miami.edu/laora/ |
| University of Wisconsin OPO | http://www.surgery.wisc.edu/ |
| Upstate New York Transplant Services, Inc. | http://www.unyts.org/ |
| Washington Regional Transplant Consortium | http://www.wrtc.org/ |
| Washington Regional Transplant Consortium  (Suburban Maryland) | http://www.wrtc.org/ |
| Wisconsin Donor Network | http://www.wisdonornetwork.org/ |
| Donate Life America | http://www.donatelife.net/ |

OPO=Organ Procurement Organization.

URL= Universal Resource Locator
